# Participatory policy analysis in health policy and systems research: reflections from a study in Nepal

**DOI:** 10.1186/s12961-023-01092-5

**Published:** 2024-01-09

**Authors:** Sujata Sapkota, Simon Rushton, Edwin van Teijlingen, Madhusudan Subedi, Julie Balen, Sujan Gautam, Pratik Adhikary, Padam Simkhada, Sharada P. Wasti, Jiban K. Karki, Sarita Panday, Alisha Karki, Barsha Rijal, Saugat Joshi, Srijana Basnet, Sujan B. Marahatta

**Affiliations:** 1 Manmohan Memorial Institute of Health Sciences, Kathmandu, Nepal; 2 The University of Sheffield, Sheffield, United Kingdom; 3 Bournemouth University, Poole, United Kingdom; 4 Patan Academy of Health Sciences, Lalitpur, Nepal; 5 Canterbury Christ Church University, Canterbury, United Kingdom; 6 PHASE Nepal, Bhaktapur, Nepal; 7 University of Huddersfield, Huddersfield, United Kingdom; 8 University of Greenwich, London, United Kingdom; 9 Liverpool School of Tropical Medicine, Liverpool, United Kingdom; 10 University of Essex, Colchester, United Kingdom; 11 Nepal Open University, Lalitpur, Nepal

**Keywords:** Participatory policy analysis, Health policy and systems research, Qualitative evidence, Nepal

## Abstract

**Background:**

Participatory policy analysis (PPA) as a method in health policy and system research remains underexplored. Using our experiences of conducting PPA workshops in Nepal to explore the impact of the country’s move to federalism on its health system, we reflect on the method’s strengths and challenges. We provide an account of the study context, the design and implementation of the workshops, and our reflections on the approach’s strengths and challenges. Findings on the impact of federalism on the health system are beyond the scope of this manuscript.

**Main body:**

We conducted PPA workshops with a wide range of health system stakeholders (political, administrative and service-level workforce) at the local and provincial levels in Nepal. The workshops consisted of three activities: river of life, brainstorming and prioritization, and problem-tree analysis. Our experiences show that PPA workshops can be a valuable approach to explore health policy and system issues – especially in a context of widespread systemic change which impacts all stakeholders within the health system. Effective engagement of stakeholders and activities that encourage both individual- and system-level reflections and discussions not only help in generating rich qualitative data, but can also address gaps in participants’ understanding of practical, technical and political aspects of the health system, aid policy dissemination of research findings, and assist in identifying short- and long-term practice and policy issues that need to be addressed for better health system performance and outcomes. Conducting PPA workshops is, however, challenging for a number of reasons, including the influence of gatekeepers and power dynamics between stakeholders/participants. The role and skills of researchers/facilitators in navigating such challenges are vital for success. Although the long-term impact of such workshops needs further research, our study shows the usefulness of PPA workshops for researchers, for participants and for the wider health system.

**Conclusions:**

PPA workshops can effectively generate and synthesize health policy and system evidence through collaborative engagement of health system stakeholders with varied roles. When designed with careful consideration for context and stakeholders’ needs, it has great potential as a method in health policy and systems research.

## Introduction

Health policy and systems research (HPSR) is highly complex. Particular complexities ensue from the dynamic (yet synergistic) relationships between policy and system, the intricate nature of health policy and system evidence [1], and rapidly changing health policy and system contexts [2]. Although recognized, these complexities remain inadequately addressed [3]. Additionally, despite increased attention to the policy component (for example, policy research/analysis) in recent years – indicated by the rapidly evolving policy analysis frameworks to address complexity [4, 5] – this has, nevertheless, been comparatively neglected compared with the systems component [6]. This neglect particularly holds relevance in low- and middle-income countries, where scarce resources/insufficient guidance have impacted effective health policy research/analysis [4, 5, 7].

Policy research seeks to address underlying social problems (in this case, problems relating to the health system) by generating pragmatic solutions and projecting their large-scale social impacts [8]. A key aspect of policy research is policy analysis, which involves systematically analysing policy or its component(s), the policy processes, comparing policy alternatives, and examining the causes and/or consequences of policy decisions to inform future actions [9–11]. These are “value laden” processes influenced by multidimensional and multidisciplinary variables subject to influence and intervention [8]; policy analysis assesses problems through various lenses and considers different disciplines to think about them [11]. Although scientific evidence has a role to play in informing policy, considerations of political climate, organizational factors and bureaucratic regulations are also key to effective public policy analysis [12]. These factors interact with each other, as well as with issues of the distribution of power between policy and system actors, and conflicting interests, ideas and values. To address the complexities associated with policy research, policy analysis has gradually shifted from its traditional, unicentral, analytic and scientific approach to a more interactive stakeholders-based approach [13]. Stakeholders’ involvement is considered vital for the production and communication of salient, credible and legitimate knowledge [14].

As a consequence, in recent years, participatory approaches have become more common in policy analysis [1, 15]. Participatory policy analysis (PPA) is one such approach. Participatory methods enable stakeholders to analyse, share, educate, plan, evaluate and prioritise specific issue(s) of interest [16]. With their origins in the 1970s, participatory approaches in general, and PPA methods in particular, have evolved significantly [13, 16]. Whilst “expert-led” policy analysis approaches have been criticized [1], PPA has been seen as a useful method [17, 18] in which researchers become “collaborators with policy setters and policy implementers” rather than external imparters of wisdom [19].

Participatory approaches to policy analysis can be used to identify and define policy problems, analyse them and provide recommendations for future action. They have been used in a variety of sectors, such as environmental and agricultural policy [20–22]. However, PPA as a method in HPSR remains underexplored. There is a need for more research to explore the usefulness of PPA in HPSR, to document potential approaches to designing and implementing PPA in the health sector, and to understand if, and how, PPA can contribute to policy in practice [13].

Our research [23], applying PPA as a primary method to explore the health reform initiated by Nepal’s move to a federal government system, offers important insights into the use of PPA in HSPR. Our choice of PPA as a central method resulted from our understanding that (a) those working within the health system are best placed to diagnose its failures and devise solutions; (b) the health system is a social system in which individual stakeholders and governance levels, and their relationships, guide its success (or, failure); (c) political/constitutional changes (such as the move to federalism) have a dynamic impact on the health system; and (d) a PPA approach can constructively engage different health system actors and encourage them to discuss and compare perspectives on the reform process, meaning that the process as well as its outcomes are seen as being of importance and worthy of analysis.

In this paper, we present our reflections on the usefulness of PPA workshops in HPSR. Although we provide illustrations/examples from our data to support our reflections, our aim in this article is not to present findings on the impact of federalism on Nepal’s health system. Rather, in this paper, we set out to answer the question “What are the strengths and challenges of participatory policy analysis for health policy and systems research?”.

We begin by providing an account of the study context and the design and implementation of the PPA workshops, followed by the associated strengths and challenges.

## Study context

Nepal’s health sector is undergoing a major reform instituted by the country’s move to a federal government structure. A centralized kingdom until 2008, Nepal has since transitioned from a republic (2008–2015) to a federal republic (2015–present). The introduction of federalism devolved power and resources from the central government to seven new provinces and 753 local governments.

Before federalism, Nepal’s health system was largely centralized. While district headquarters oversaw local health facilities, the supervision was guided by the central government with a clear “chain of command” (from central to regions to zones to districts to local levels). Nepal’s 2015 constitution divided health system functions between the three levels of government (federal, provincial and local), with some shared and some sole functions assigned to each level of government [24]. While policy-making powers are divided between the federal and provincial governments, local governments have responsibility for the provision of basic health services – and are thus important policy implementers [24].

Federalism in Nepal has been a result of political grass-root reaction against the long-standing centralized system, and it has not progressed through a systematic evolutionary process nor have its mechanisms been guided by any research [25]. The process therefore is facing several challenges, including a lack of cooperation and overlap/confusion in the functions and responsibilities among the three government levels; service delivery interruptions; and problems with resource mobilization, financial autonomy, effective participatory planning, budgeting and implementation at the local level [25–27].

With the transition to federalism still a work in progress, Nepal’s health system stands at a critical juncture. To steer the system in the right direction, policy-makers and health managers need quality information and analysis. Continuous appraisal of health system development, and systematic investigation to provide evidence to inform and strengthen health reform policies, processes and mechanisms are required. Stakeholders, including health service providers, policy-makers, development partners and health system researchers need to come together and collaboratively participate. We designed the workshops to provide a “platform” for health system stakeholders (including health workers, administrators, (International) Non-Governmental Organizations ((I)NGO)/development partners and policy-makers) to come together and, through collaborative discussions, explore the effects of the country’s move to a federal system on their work and the new challenges it has created; personally, collectively and for the health system.

## Design and implementation of participatory policy analysis workshops

### Study sites

Twelve PPA workshops were conducted (including one pilot) across five districts (Nawalparasi West, Sindhupalchowk, Mugu, Kathmandu and Lalitpur) in Nepal, purposively selected to represent the country’s three ecological regions (plains, hills and mountains) and to include the federal capital. The pilot workshop was conducted in Lalitpur district. Within each study district, two municipalities (representing a rural and an urban setting) were purposively selected. Including the pilot, these workshops covered nine local governments (four municipalities, two metropolitan cities and three rural municipalities) and three provincial governments (Bagmati, Lumbini and Karnali provinces).

### Study participants and recruitment

Health system stakeholders with wide-ranging roles participated in the workshops. The desired mix of roles was determined in advance by the research team, to include (a) stakeholders who could make or influence health and health system related decisions (such as elected political leaders), (b) local and provincial health administrators leading or representing key health offices/units or departments (such as health ministry, health directorate, municipality health department, hospitals/health posts, planning commission), (c) health service providers (for example, auxiliary health workers [AHW], clinical and nursing staff, auxiliary nurse and midwife [ANM], female community health volunteers [FCHVs]) and, where relevant, (d) external development partner representatives (such as from (I)NGOs) working closely with the local or provincial governments and the health system.

The research team approached the municipality health departments at the local level, and ministries overseeing health at the provincial level, in most cases through the person(s) in charge, such as the spokesperson or the head of the department/section (referred to as “gatekeepers” from here on), and discussed with them the research project and the details of the PPA workshops. We provided them with a list of health system stakeholders categories/roles that we wanted as workshop participants (as represented in Table 1). To ensure gender balance, the research team specifically requested the gatekeepers identify at least 33% and, where possible, 50% female participants in the stated roles.

**Table 1:** Workshop participants

Local level		Provincial level	
Participants	Number (gender)	Participants representing	Number (gender)
• Elected representatives (for example, mayor, ward chairs, vice chair and ward members)	17 (12 M, 5 F)	• Province health ministry (or equivalent)	13 (10 M, 3F)
• Ward and municipality administrators (for example, ward secretary, acting/chief administrative officer)	7 (M)	• Province health directorate	6 (5 M, 1F)
• Municipality health administrators (for example, health coordinators, others working in the municipality health department):	15 (11 M, 4F)	• Province/district hospital/health centre	6 (4 M, 2F)
• Service providers (for example, ANM, AHW, doctors, those working in health facilities and/or engaged in service delivery including FCHVs)	33 (17 M, 16 F)	• Province health training centre and related	3 (M)
• External development partner representatives	2 (M)	• Provincial logistic management:	3 (M)
		• Provincial public health laboratory	2 (M)
		• District health office	3 (M)
		• Province planning commission	1 (M)
		• External development partner	3 (M)
Total (11 workshops)	74 (49 M; 25F)		40 (34 M; 6F)

*ANM* auxiliary nurse and midwife, *AHW* auxiliary health worker, *FCHVs* female community health volunteers, *M* male, *F* female

Based on the list of roles provided by the researchers, individual workshop participants, in most cases, were identified by these gatekeepers. The researchers then contacted the participants to provide information about the workshop. Where researchers could not make direct contact, the gatekeepers contacted the participants. In many cases, the gatekeepers were also themselves participants in the workshops. Workshop times and venues were decided in consultation with the gatekeepers to maximize the availability of participants and minimize the potential impact on routine health service delivery.

We had previously conducted key informant interviews with health system stakeholders in the study sites. The researchers, therefore, had had prior contact and had developed relationships with the stakeholders/gatekeepers. This facilitated effective collaboration and organization of workshops. Participants numbers varied among workshops, and ranged from 7 to 15. A total of 74 stakeholders participated in local-level workshops and 40 in province-level workshops (Table 1).

### PPA workshop: process and activities

We designed PPA workshops consisting of three main activities, namely (1) river of life (RoL), (2) brainstorming and prioritization and (3) problem tree analysis. The activities were chosen because they were able to delve into the individual and collective experiences of the health system stakeholders, they were not “too complex” for the participants and they would foster participative engagement. The selected activities are all well-established formats and have been used to explore social and health issues [28–31]. We sequenced these activities to lead participants from their individual experiences of health system change – getting them thinking about the ways in which federalization had impacted on their working lives (RoL), to thinking collectively and exchanging ideas about the challenges resulting from federalization (brainstorming and prioritisation), to thinking about the root causes of those problems and potential solutions (problem tree). Table 2 summarizes the activities, their objectives and how each activity was conducted (the processes).

**Table 2:** PPA workshop activities details, objectives and processes

Activities	Description	Objectives	Process/data
River of Life (RoL)	An art-based autobiographical mapping (reflective) tool that encourages group members’ bonding, RoL uses the metaphor of river to depict a personal journey or history as well as future aspirations. River bends, width and discontinuities represent the junctures, breadth of perspectives, events and pauses that have occurred during the life course. RoL can be used alongside other approaches to explore particular issues and problems [28, 32] and is especially useful for understanding change over time	(a) Get each participant thinking about federalism and its impact on the health system and their working lives (b) Understand the impacts of federalism on health system stakeholders’ working lives (c) Explore stakeholders’ perceptions of how the health system, their journey and experiences in the health sector have changed over time (focus on before and after federalization)	*Participants sketched on large chart paper the “flow” of their working lives and their interaction with the health system, with a focus on changes that have resulted due to federalism* *They then presented their “rivers” to the other participants in the workshop*
Brainstorming and Prioritization	Brainstorming—a creative thinking process—is the most common and well-known technique for ideation, increasing creative efficacy or for finding solutions to problems. It can be conducted as an individual or group exercise. Prioritization involves collaborative discussion to prioritise issues that have arisen through the brainstorming based on their perceived order of importance. While brainstorming is a divergent phase aiming for quantity (numbers of ideas/issues), prioritizing is the convergent phase where there is sorting of the ideas/issues identified to find those that are judged to be most crucial [33–36]	(a) Get participants thinking about the full range of problems and challenges brought about by federalization, and how those problems have affected the health system and/or their own working lives (b) Collaboratively prioritise federalization-related policy/programme issues	*Participants were grouped into random groups using the chit method. Each group brainstormed as many problems/issues as possible. This was followed by rigorous discussion in groups to prioritise the identified problems. Participants considered issues that they felt were creating significant impacts on the health system and/or their own working lives*
Problem Tree	A “tree” diagram showing the cause–effect relationship between problem conditions in a defined context. Problem tree analysis thus involves working through a defined problem to map out the cause and effect surrounding the problem, which in turn leads to finding/ investigating solutions, either through tackling root causes or seeking to mitigate effects. By allowing the prioritized problem to be broken down into manageable and definable chunks, it enables clear understanding of the problems and its often interconnected and sometimes contradictory causes [37, 38]	(a) Collaboratively create a “problem tree” – to identify root causes and consequences of the group’s chosen problem (identified in the previous stage) (b) Work together to develop relevant policy or practice suggestions to resolve the problem or mitigate its effects	*Each group collaboratively developed a problem tree for the key problem they identified in the brainstorming and prioritization session. The problem was represented by the trunk of the tree, the multiple causes of the problems as roots and the consequences – small and large – as branches of the tree. The problem analysis was followed by group discussion of possible solutions/ mitigations (at a practice and policy level)*

### Data collection and analysis

Data were collected in different forms: activity chart sheets, contemporaneous notes taken by note takers during the workshops and field notes written by the facilitators. To ensure that participants felt free to share their views openly, and to enable free-flowing and interactive discussions, it was decided not to audio- or video-record the sessions, which the research team felt would have negatively altered the dynamic. The research team collected all the activity chart papers generated from each activity: the RoL, brainstorming and prioritization, and problem tree (Table 2). Note takers were exclusively engaged in taking detailed notes of the workshop discussions and presentation contents. The facilitators also made fieldnotes – of their observations and interactions. To uphold consistent and precise data collection, the “notetaking” aspect was discussed in detail, prior to and after the pilot PPA workshop. The (already experienced) facilitators and note takers were also upskilled on the selected workshop activities and sensitized to the possible issues (such as gender and power dynamics) that could arise during the workshop.

As part of the PPA process, the first-level analysis was conducted by the workshop participants themselves. The participatory nature of the workshops enabled a form of “data validation” within the workshops themselves as participants and small groups presented their perspectives and analyses, and these were then discussed, reflected upon and, in some cases, challenged by other participants in the workshop. The contemporaneous notes taken by the research team captured these discussions. Second-level analysis by the researchers used collective presentations/summaries of the first-level analyses of data from all sites, and content analysis of the material generated by the participants themselves (for example, the RoL and problem tree drawings) and the notes taken by the research team. Data collected in different forms were triangulated to ensure accurate analysis. We also discussed the key draft findings with the wider international project team to have a wider range of perspectives and improve interpretation. The findings from these analyses will be presented in subsequent publications reporting the findings.

## Opportunities and challenges of PPA in HPSR

PPA, we have found, offers great value in exploring health policy and system issues and has advantages beyond the generation of reflective and rigorous qualitative data. PPA workshops can bridge the gap between understandings of political and practical/technical health system aspects and bring forth issues that remain hidden to stakeholders holding different roles. Gathering together different health system stakeholders and engaging them in the policy analysis process, however, comes with challenges (Table 3). For effective outcomes, stakeholder engagement and influence need to be carefully navigated.

**Table 3:** PPA workshop method: opportunities and challenges

Opportunities	Challenges
• Context suitability and acceptability	• Stakeholders’ influence on the process
• Possibility to generate comprehensive qualitative evidence	• Influence of power and literacy dynamics
• Ability to bridge gaps in understanding of practical/technical and political aspects of health/health system	• Raised expectations of researchers’ ability to address issues
• Applicability beyond “data collection” – PPA as intervention	

### Opportunities

#### Context suitability and acceptability

PPA workshops to explore federalism’s impact on the health system received a positive response and enthusiastic support from all participants and gatekeepers. In addition to supporting the workshops, key gatekeepers (who were also in most cases workshop participants) from the municipal and provincial health departments took ownership of them, facilitating workshop organization, easing researchers’ access into the system and positively influencing participants’ commitment to the workshop.

The contextual factors that we consider important in explaining the acceptability and success of the PPA workshops in Nepal are threefold. Firstly, federalism is a current trending issue in Nepal in which there was considerable interest from all stakeholders. Secondly, democratic discussions to cultivate intellectual and productive interactions as a regular part of political or health system-level decision-making in Nepal are not a norm. Policy-related discussions in Nepal (as in many other countries) usually only involve people at the political level and exclude the ground-level workforce. Thirdly, although hierarchies are a strong feature of society (as discussed further below), Nepal is a “liberal” society in which it is possible for people to have political discussions openly and to disagree with and criticize government without fearing for their safety or job security. Such workshops might not be possible in more authoritarian political systems.

Political relevance, timing and the degree of controversy surrounding the social and policy issue under discussion are key factors that facilitate meaningful and successful participatory (policy) approaches. Engendering enthusiasm for less interesting topics or issues that are not presenting some kind of social, environmental or political dilemma within a society is difficult [39]. Stakeholders clearly felt a need for a platform to discuss issues surrounding federalization and health. This resulted in participants’ enthusiastic and active engagement in the workshop activities.

“It’s been two years and they haven’t organized a single meeting with our Mayor, administrative leaders or us – the health people”. (Local-level workshop – municipality Health Official)

#### Possibility to generate comprehensive qualitative evidence

PPA workshops as a research method can provide insight into both broader and specific issues of the health system and can generate rich qualitative evidence. Besides the context, stakeholders’ engagement and the process/design are crucial to PPA’s ability to generate evidence.

Interactions between diverse health system stakeholders – from elected (political) representatives to health administrators and health service providers, including community health volunteers – generated comprehensive data from a wide range of perspectives on Nepal’s health system. In our study, the workshops inspired discussions on the contemporary health system and political issues from multiple perspectives and surfaced areas of both agreement and conflicting views on the system.

PPA offers flexibility in data collection. The workshop modality, in particular, provides the opportunity to utilize different activities to generate data in desired formats and various levels of richness, and to draw on the strengths associated with each activity [40, 41]. Our interactive workshops provided creative settings for participant interaction and the co-creation of concepts/ideas and policy recommendations. Additionally, they provided an opportunity for the researchers to take contemporaneous field notes whilst observing participants as they interacted and negotiated. The ability to customize the activities to suit the research questions, context and participants was particularly important considering the novelty of the process in the setting it was being conducted. The three activities allowed in-depth discussions and the generation of rich and varied data and facilitated the following:

##### Understanding of stakeholders’ perceptions on health system change over time

The river of life (RoL) activity generated information on participants’ “personal journey within the health system”, bringing into focus their successes, struggles and changing expectations over time, and provided an insight into their personal stories. Participants had a chance to contemplate the past–present–future prospects for the health system. One participant said that the workshop provided an opportunity to reminisce about past experiences, and to reflect on how the situation has evolved over time.

“[The] most interesting thing [for me has been that] I got to recall my past experiences. The [change following federalism at the] beginning was hard to handle- but now I find it very nice to share here how things have evolved – ‘did I even have such days?’” (Local-level workshop – nurse)

**Fig. 1:**
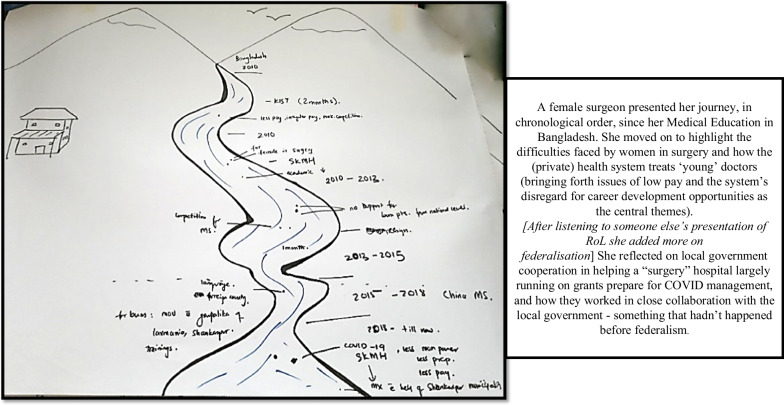
River of life depicting a participant’s journey in the health sector

**Fig. 2:**
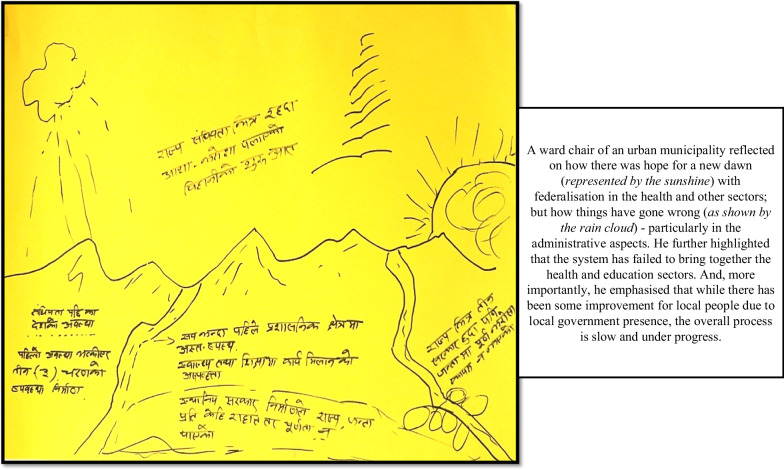
River of life depicting system issues only

**Fig. 3:**
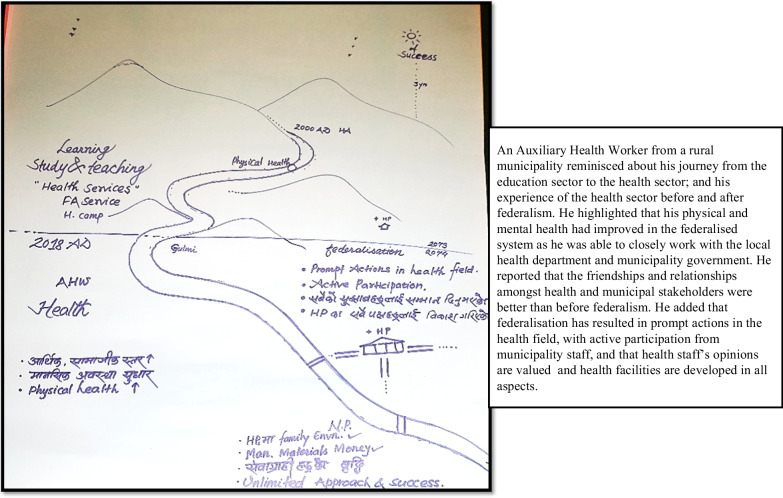
River of life where the participant combines health system issues with their personal journey

Some participants focused specifically on their own career journey (Fig. 1), some more on the “journey of the system” (Fig. 2) and others combined the two, providing data addressing different dimensions (Fig. 3).

The RoL activity thus inspired deep reflections, provided in-depth insights into stakeholders’ professional and personal lives, and allowed for creative expression when some added illustrations that they felt were helpful to tell the “story” (Fig. 2).

##### Identification of health system problems from multiple perspectives

Effectively conducted brainstorming and prioritization sessions are invaluable in identifying issues and forming a consensus among diverse groups [35]. The random allocation of stakeholders into a subgroup for brainstorming in our workshops brought people with different roles into discussion; for example, pairing a ward chair with a health post in charge facilitated empathetic understanding and engagements and allowed a “hard” discussion to take place.

**Fig. 4:**
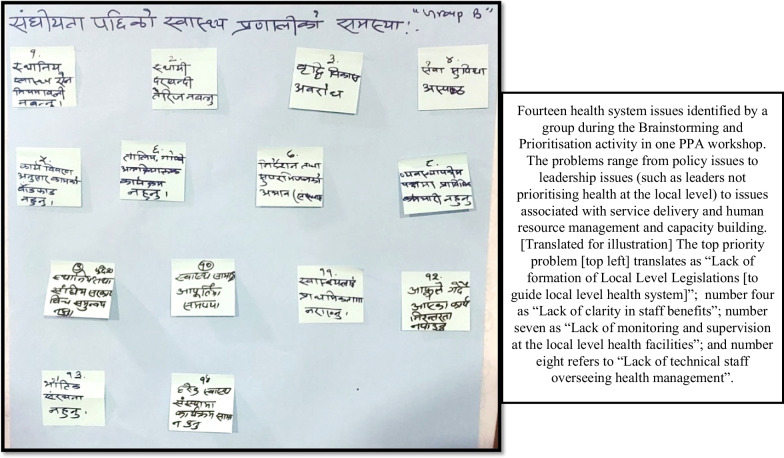
Brainstorming and problem prioritization activity – group output

The activity brought forth wide range of health system issues, ranging from leadership/administrative issues to problems related to community-level service delivery. Collaborative prioritization provided insights into differing perspectives on the most pressing problems of the health system, the resolution of which was considered important by health system stakeholders of different roles (Fig. 4).

The random allocation of participants to subgroups worked well in our study as the subgroups were small. However, if subgroups are larger (where random allocation could lead to some subgroups including more than one individual with similar job roles/traits), allocating individuals to groups non-randomly (for example, using a quota sampling method) could be useful to ensure that stakeholders with different roles are included in subgroups.

##### Analysis of problems and exploration of potential solutions

**Fig. 5:**
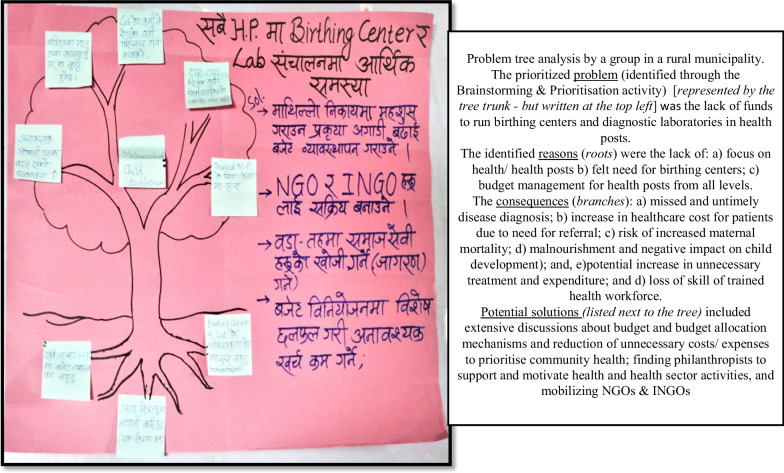
Problem tree analysis by a group in a rural municipality

The problem tree analysis generated data on health system stakeholders’ perceptions of the underlying causes and impacts of the prioritized health system-related problems and their potential solutions (Fig. 5). Across 12 workshops, a wide range of problems that the health system is currently facing, as well as their potential solutions, could be explored.

PPA workshops allowed for the collection of data in different forms, ranging from notes (from the interactions/discussions), field notes based on researchers’ observations of the interacting groups’ dialogue and discussions, and the materials generated in the workshop – drawings, discussions and models.

#### Ability to bridge gaps in understandings of practical/technical and political aspects of health

PPA workshops can effectively bridge existing gaps in practical/technical and political issues and perspectives; for HPSR this equates to the divide between health and politics.

Despite the highly technical nature of health and healthcare, health system reform amidst federalism is inherently a political process, and analysis should consider both political and technical aspects associated with health and the health sector. Only through the effective mix and engagement of both technical and political health system stakeholders can health reform issues be effectively explored.

Two aspects of PPA workshops are key in addressing the technical/political gap. First, the workshops can bring technical (for example, health service providers or health system bureaucrats) and political role holders (for example, elected representatives) together. Second, PPA can bring practical/technical and political issues into the discussion, and allow participants from both sides of the divide to understand the priorities, incentives and constraints of the other.

In Nepal, there are increased expectations on local elected leaders and administrators who, in the new federal scenario, have the ability/responsibility to make decisions that have significant impacts on the health system. Their interests in, and understanding of, the health sector/system has the potential to affect how local-level government, and even the community itself, prioritizes health. Their understanding and awareness of the problems that the health system is facing can help ensure informed decision-making. Despite this, the political and health stakeholders mostly work in silos. PPA workshops brought them together, in many cases for the first time, and initiated discussions that brought forth technical and administrative as well as political issues in a single platform. PPA was recognized as helpful by the elected leaders and municipality administrators – who often do not closely work with health professionals but do contribute to health-related decisions. For example, one group of workshop participants identified “improper management of health staff at local level” as a key problem post-federalization. The issue was discussed in depth within the group, during which the municipality administrative officer – a non-health person – responded:

“I never thought this was a [significant] problem – but I can now see that this is!” (Local-level workshop – municipality, Chief Administrative Officer)

At the end of another workshop, a ward-chair (an elected political member) shared what he learnt from the workshop:

“This workshop is different. We have been able to have focused discussions about health in the federalized context in our municipality. It has become clear [from the discussions] that we haven’t been able to do enough (on health); and, that there is frustration. For solutions, we are lacking a bold team- this workshop has highlighted the problems, and how to solve them. It has highlighted the need to prioritise health. I will put this forward: that in health, budget should be adequately allocated and spent. It has made us aware of our misplaced priorities in the current situation. You have given emphasis on [the need for] quality over quantity [in the health sector]- thank you. We anticipate feedback from the [health] team for our municipality. I will also raise these issues in meetings…” (Local-level workshop – Ward Chair)

Another workshop had a ward member who had met the health staff working in his ward for the very first time in the workshop:

“I got to learn about the problems that our health staff are going through. They are facing such problems. It’s good that I got an opportunity to meet you all – the staff of this municipality as well”. (Local-level workshop – ward member)

Meanwhile, the PPA activities helped health staff obtain an understanding of the roles and limitations the leaders and administrators had. The health staff also took this as opportunity to put their views and points in front of administrators and elected members – utilizing the rare chance to discuss issues they felt often went unheard.

“Your objective is research [data]… [but] our intention [here] is also to put the issue to the concerned authority [referring to the Chief Administrative Officer of the Municipality]” (Local-level workshops – Municipality Health Official)

This helped in creating links and clearing up misunderstandings, and thereby strengthening the relationships between the stakeholders at the local level – something we feel is crucial for collaborative engagements and development in future to garner often lacking political commitment to health system improvement [42]. At the same time, these conversations were not always easy. Nepal is still a “hierarchical society”, where it is difficult for “juniors” to openly speak in front of seniors or those occupying more powerful positions. The PPA workshops as a “research activity”, however, provided something of a “neutral platform” wherein they could express their views more openly. In a different setting, for example, a “political” event, this could have been a more tense environment between stakeholders who viewed the health system issues differently.

For HPSR, therefore, PPA workshops can become platform for health and political actors to discuss, envision and unravel competing interests and goals, and to build mutual understanding.

#### Applicability beyond “data collection”

Participatory methods, such as PPA are, by their nature, “interventions in society” [39]. PPA workshops can act as a health system strengthening intervention with shared learning as a primary component. Their applicability beyond data collection is twofold:

##### Relevance to the workshop participants

PPA workshops can enhance participants’ understanding of the contemporary issues surrounding the health system and can, therefore, be “educational”.

“[Through] the participatory modality … [we have learnt collectively] how we are working, what we are doing. There are a lot of things that my mind is thinking about right now. My mind has been sensitized. What we lack, what we should do and all”. (Provincial-level workshop – Sr ANM, District Hospital)

Its ability to bridge the divide between politics and health system practice, as explained above, was largely because participants could learn the perspective of the “other side”.

##### Relevance to the health system

PPA workshops are able to break down the divide between data collection and policy dissemination. Policy dissemination involves the distribution of evidence produced through research to policy-makers, to promote understanding on the adoption and sustainment of evidence-based policies [43]. For policy dissemination, it is important to understand different policy systems and players. Identifying policy elements that are and are not likely to be effective is crucial not only for the policy-making process, but also for how policy is implemented and how issues in implementation are addressed [43]. Our workshops brought together policy-makers and those who implemented and were impacted by the policy in the same room, creating an environment for the sharing of first-hand experience with policy audiences.

Additionally, PPA workshops are able to generate new practice and policy recommendations for the health system. In alignment with policy dissemination research, our workshops focused on identifying and understanding the problems the health system is facing in the federalized context, and exploring mechanisms to solve them [43]. It created a platform wherein problems (and solutions) were identified and communicated directly to the “policy-makers” by the “policy-implementers”. Workshop modalities are deemed useful in dissemination [44]. The problem tree analysis activity, in particular, was significant in this regard; it aided detailed analysis of the health system problems and the identification of potential solutions (Fig. 5). The participants identified barriers ranging from health staff shortages to a lack of effective policies to guide the health system in a federalized context. In some cases, the administrators who were present in the workshop had a holistic understanding of the wider political environment, which facilitated exploring the possible solutions, their mechanics and implementation. Solutions that ranged from small-scale, practice-level changes with potential for prompt implementation to broader policy-level changes were identified through PPA (Fig. 5).

### Challenges

#### Gatekeepers’ influence on the process

Gatekeepers’ (who were often also workshop participants) interest in the workshop, although a key factor influencing participant engagement, also brought challenges. Acceptance and ownership by gatekeepers had an influence, to some extent, on who participated in the workshops, when they were conducted and for how long. Occasionally, the workshops had to be scheduled at short notice, depriving the researchers of effective prior contact with the workshop participants.

A major influence was seen in the number and gender of the participants. Despite always communicating that we needed eight to ten participants per workshop and ideally an equal mix of male and female participants, some workshops had as many as 15 participants. While the group size did not impact the research findings per se, the smaller groups were easier to manage and allowed in-depth discussions to take place more easily within the stipulated time.

In some cases, there was a severe gender imbalance. Except in one workshop which had seven (out of eight) female participants, in all others male outnumbered female participants. Overall, only 33% of participants in the local-level workshops and 15% of participants in the provincial-level workshops were female (Table 1). While this could be a representation of the male domination of the political, administrative and health arenas in Nepal (especially at senior levels), this was also influenced by the gatekeepers who selected the participants. Engaging gatekeepers in the participant identification and recruitment process, although helpful, imposed limitations by creating gender imbalanced groups, resulting in situations where the voices and opinions of female stakeholders may not be effectively represented.

#### Influence of power and literacy dynamics

Stakeholders’ engagement in any participatory process should be handled cautiously in light of the power and gender dynamics between participants, and activities should be effectively designed to take into account the backgrounds, make up and personalities of participants [45, 46]. Power dynamics and domineering engagements of some stakeholders, usually of those who sit on the higher rungs of the political or bureaucratic system, has the potential to sway the discussions in the direction they choose, thereby suppressing the voices of stakeholders from “lower” levels in the system, such as health service providers [45].

Although these issues were taken into consideration in the workshop design phase, and facilitation sought to further ensure that power dynamics did not distort the process, they could not be completely avoided. The power of elected representatives and chief administrators was evident in the workshops. While these dynamics did not, we felt, have a major negative influence, they did add some “formality” to the process, and particularly influenced the engagements of the FCHVs. The challenge for the FCHVs is fuelled by their lower level of literacy and the informal nature of their employment, which places them “outside” formal arrangements [47]. In a few cases, the workshop facilitators observed FCHVs sitting quietly in a group where the other members were health staff and elected officials. They needed a nudge, and sometimes help, to put their thoughts on paper. Further, although in the brainstorming sessions we could see that the group listed issues suggested by FCHVs, frequently they were not prioritized and other issues took precedence. In a few cases, we were culturally obliged to invite political and administrative leaders to fulfill formalities (for example, to open the session and give a formal speech). While they sometimes provided valuable insights, they did not fully participate or left early, and undoubtedly had an impact on the power dynamics in the room.

In some instances, what might be thought of as “problematic” results of power dynamics within the room actually had unexpected positive benefits. For example, we found that a small number of participants in senior positions were reluctant to do drawings/writing as part of the workshop activities, and instead asked people in more junior positions to do that. Whilst undermining the engagement of the more senior participants, in practice this allowed the more junior person to insert and present his/her own views to the group.

Power, gender and literacy dynamics are deeply rooted social issues that cannot be completely excluded from a workshop setting. Nevertheless, vigilant and experienced facilitators can ensure that their negative impacts can be reduced, for example, by inviting comments specifically from the less powerful and/or facilitating in a way that prevents domination by particular individuals. In participatory workshops, the skills and experience of the facilitators plays vital role – particularly in ensuring that all voices are heard and in effectively navigating the discussions.

#### Raised expectations of researchers’ ability to address issues

The principal behind PPA workshops was that the health system insiders brought their expertise and experiences, and the researchers “facilitated” the discussions and data generation. While this modality was embraced and researchers’ role was largely confined to introducing the topic and keeping the activities on track with limited intervention and careful consideration to not unduly influence the process, there was nonetheless an expectation on the researchers from the participants to address the issues identified in the workshops.

“We hope the problems discussed will be reported to the (relevant) authority. Hope we get positive feedback about federalism in future”. (Local-level workshop – Official, rural municipality)

“[to the researchers] I feel you have fulfilled your objective of the research; but whether and how you will implement what you have learnt here for betterment is up to you. We have told you the reality- now it’s your responsibility”. (Local-level workshop – municipality Ward Member)

While such high expectations of researchers can be anticipated, it is vital to keep those expectations realistic. Researchers conveyed to the participants their own limitations and what realistically they could do to address the issues generated through discussions, for example, disseminating the findings at different levels as a mechanism to initiate that change [48]. Importantly, the research team discussed with the participants how participants themselves might take the ideas and issues forward on a local level. However, neither dissemination of the findings or discussion with the participants can guarantee policy changes. Our research, although funded, is not an “intervention”, and cannot directly bring about change. PPA’s potential as intervention for improved communication and engagement discussed earlier can, nonetheless, be explored and exploited to “bring changes” in future.

## Strengths and limitations

This paper sheds light on the use and value of PPA in HPSR—an area where PPA remains underexplored. It offers important insights into PPA workshops, including design considerations and the opportunities and challenges PPA presents in exploring health reform in a rapidly transitioning setting. Our reflections, we believe, are useful not only to HPSR researchers and those interested and engaged in participatory methods, but also to policy-makers and implementers of health programmes. Our experiences offer insights into how PPA methods can be tailored to engage stakeholders and explore health system and policy issues and develop potential new solutions.

In utilizing the information presented in this paper, however, the following limitations should be acknowledged:

- The stakeholders engaged in the workshops were from the same government level. For example, PPA workshops were conducted separately with the stakeholders at a local level and provincial level. Furthermore, high-level politicians were not a part of this series of workshops. Hence, although our study sheds important insights on stakeholder engagement and dynamics, it is unclear what would happen if we were to bring stakeholders from very different levels together. Additionally, it is possible that policy recommendations made by stakeholders from the same (government) level may not take account of the health system context in its entirety. Also, it is easier for stakeholders to blame or put responsibilities onto stakeholders of another level not present in the workshop. Bringing together stakeholders from different levels would enable discussions between levels about the feasibility of recommendations. Further research combining stakeholders from different levels and engaging high-level politicians to discuss on health reform issue is therefore needed.
- Our reflections on PPA’s potential applicability in HPSR are based on our experience and the stakeholders’ feedback. The assessment is thus “short-term”; and, although PPA appears to have had some impact in terms of stakeholders’ engagement, it is unclear how long lasting these impacts would be. Follow-on research is required to understand this. Ideally, PPA workshops could be repeated on a regular basis. However, to effectively conduct PPA requires focused dedication and investment of time, money and effort. Designing the PPA workshops, bringing a number of different stakeholders together, facilitating the workshops and analysing the data generated are all resource-intensive processes. Whether health system stakeholders would do this without the prompt (and work) from the research team is unclear. To conduct this as a routine health system activity, however, is possible; it would require training the stakeholders on PPA and for them to take the initiative.

## Conclusions

PPA has a huge potential in HPSR, especially in generating and synthesizing health policy and system evidence, and in creating new ideas for change from stakeholders who are normally excluded from the policy process. As it continues to evolve in its approach and applicability, PPA offers HPSR researchers the ability to tailor the process to specific contexts, to effectively engage stakeholders and to address complexities associated with HPSR evidence when studying a major health reform.

PPA workshops are typically effective in exploring health system policy and practice issues from the perspective of those involved in the system, and for understanding the complexity and challenges of their everyday working lives. Our experience underscores that an appropriately designed PPA process can effectively bridge the gaps in understanding and association between practical and political aspects of health and health system, a crucial but often neglected issue in health policy and health system reform discussions. By sensitizing technical health experts to political issues and politicians to (health/healthcare related) technical issues, PPA workshops are not only able to produce rich qualitative evidence, but can also provide a platform for shared learning. The method, therefore, has the potential to be adapted as an intervention to promote communication and engagement between stakeholders of different types. In addition to having an impact on the participants, they aid in policy dissemination and produce useful practice and policy-related ideas to strengthen the health system and mitigate health system challenges. However, for an unbiased generation of data using a PPA approach, stakeholders’ roles, influence and the power dynamics of the group needs careful management. Facilitators’ understanding of the context/culture and their experience in working with health system stakeholders and conducting discussions, and their capacity to understand these dynamics and navigate them effectively, are the key factors for success.

## Data Availability

The data that support the findings of this study are available on request from the corresponding author, SR. The data are not publicly available due to containing information that could compromise the privacy of research participants.
